# RNA regulation by Poly(ADP-ribose) polymerases

**DOI:** 10.1016/j.molcel.2015.01.037

**Published:** 2015-06-18

**Authors:** Florian J. Bock, Tanya T. Todorova, Paul Chang

**Affiliations:** 1 Koch Institute for Integrative Cancer Research, Massachusetts Institute of Technology, 500 Main Street, Cambridge, Massachusetts 02139, USA; 2 Department of Biology, Massachusetts Institute of Technology, 500 Main Street, Cambridge, Massachusetts 02139, USA

## Abstract

Post-transcriptional regulation of RNA facilitates the fine-tuning of gene expression. It occurs through multiple pathways that include the nuclear processing of mRNA and its precursors, mRNA silencing, regulation of mRNA decay, and regulation of translation. Poly(ADP-ribose) polymerases (PARPs), enzymes that modify target proteins with ADP-ribose, play important roles in many of the RNA regulatory pathways through multiple mechanisms. For example, RNA-binding PARPs can target specific transcripts for regulation, ADP-ribosylation of RNA-regulatory proteins can alter their localization, activity or RNA-binding, and non-covalent interactions of RNA-binding proteins with poly(ADP-ribose) can affect their function. In addition to regulating RNA during non-stress conditions, PARPs mediate RNA regulation during cellular stress conditions that are critical for the proper execution of a stress response. In this review, we summarize the current knowledge regarding PARP-dependent regulation of RNAs, and describe how by modulating RNA processing, translation and decay, PARPs impact multiple processes in the cell.

## Introduction

Poly(ADP-ribose) polymerases (PARPs) use NAD^+^ as substrate to modify target proteins by attaching ADP-ribose. Targets proteins are either mono(ADP-ribosyl)ated or modified with long polymers of poly(ADP-ribose) that play additional regulatory functions by binding and recruiting proteins through non-covalent interactions (Bürkle, 2005; Gibson and Kraus, 2012; Vyas et al., 2014). In some cases, this binding alters the function of these poly(ADP-ribose) interacting proteins. The most studied PARP functions involve DNA regulation (Messner and Hottiger, 2011), however PARPs are also involved in key steps of RNA processing that occur in the nucleus and the cytoplasm (Ji and Tulin, 2013). This is somewhat ironic since PARP activity was initially thought to result in the synthesis of poly(A) RNA, not the molecule we now know as poly(ADP-ribose)(Chambon et al., 1963). In this review we summarize known PARP functions in the regulation of RNA, describing key PARP-dependent regulatory steps beginning with the initial processing of pre-mRNA and rRNA in the nucleus and ending with regulation of mRNA decay in the cytoplasm. We discuss known roles for PARPs in RNA regulation during stress and show that, for the most part, these appear to be extensions of physiological, non-stress functions of PARPs.

**Figure 1:**
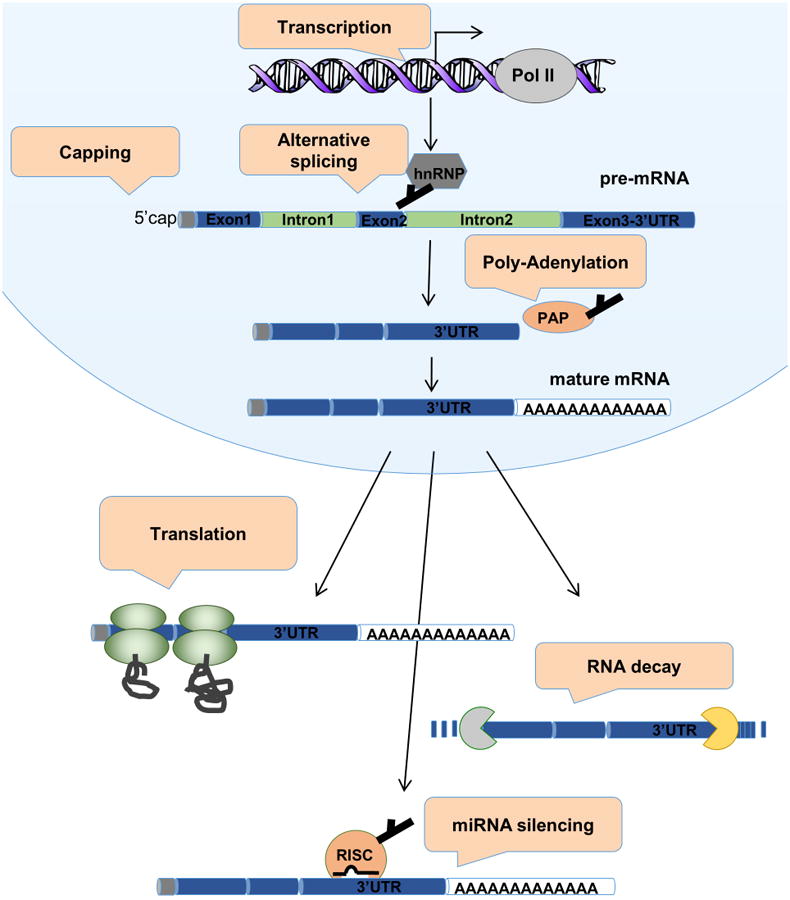
The RNA life cycle RNA is transcribed in the nucleus, after which intronic sequences are removed by splicing, a methyl-guanine cap is added to the 5′ end and a poly(A) tail is attached at the 3′ end (figure is not meant to depict an exact order of events). After export to the cytoplasm the mature RNA can be translated by ribosomes, degraded by RNA decay or silenced by microRNAs.

In eukaryotes, mRNAs, initially transcribed as pre-mRNAs, are processed in the nucleus before the resulting mature mRNAs are exported to the cytoplasm (Figure 1). Nuclear processing of pre-mRNAs involves capping with a methyl-guanine cap at the 5′ end, removal of non-coding introns by splicing, and cleavage and poly-adenylation at the 3′ end. These modifications increase stability and facilitate export of the now mature mRNAs from the nucleus (reviewed in (Hocine et al., 2010; Manley, 2002; Moore and Proudfoot, 2009; Proudfoot et al., 2002)). Once in the cytoplasm, mature mRNAs are either translated into protein, silenced by microRNAs, or targeted for degradation in a process called RNA decay (reviewed in (Fabian and Sonenberg, 2012; Garneau et al., 2007; Jackson et al., 2010; Roy and Jacobson, 2013; Schoenberg and Maquat, 2012; Spriggs et al., 2010; Wilson and Doudna, 2013). Nearly every step of this complex life of the mRNA is regulated by PARPs and the ADP-ribose modifications they generate.

## The important players

RNA binding proteins (RBPs) assist in each step of RNA metabolism and are the most important class of proteins that regulate RNAs. They are targeted for modification by PARPs, and in some cases their function is altered by non-covalent binding to poly(ADP-ribose). In addition, several PARPs are themselves RBPs and contain well-defined RNA binding domains.

### PARPs that are RNA binding proteins

**Figure 2:**
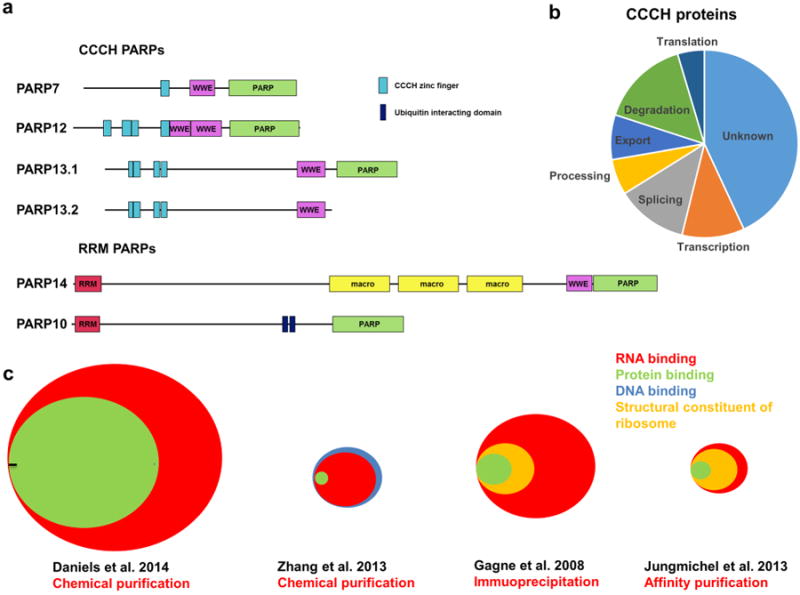
The role of PARPs in RNA binding (a) Schematic diagram of PARPs that contain RNA binding domains. PARPs are drawn to scale to highlight similarities in the spacing and length of CCCH zinc finger domains. (b) Analysis of all human proteins that contain CCCH domains. Function of individual proteins was classified into the indicated categories. Proteins containing these domains function in nearly all aspects of RNA biology. (c) Functional classification of ADP-ribose associated proteins identified in proteomic studies. The type of ADP-ribose purification method utilized in each study is shown in red. Proteins identified in these studies were analyzed by GO term analysis, and categories relevant to RNA biology shown. The size of the circle is inversely correlated with the p-value of the enrichment, e.g. the smaller the p-value the larger the circle. Scale bar in the first circle represents a p-value of 1^-10^.

There are five PARPs that can be defined as RBPs. These include PARP7, PARP12 and PARP13 that contain RNA binding CCCH zinc finger domains, and PARP10 and PARP14 that contain RNA recognition motifs (RRM) (Figure 2A) (Vyas et al., 2012). In addition PARP2 contains a less defined SAF-A/B, Acinus and PIAS (SAP) domain shown to bind rRNA *in vitro* (Léger et al., 2014). RNA-binding PARPs are unable to ADP-ribosylate or alter the RNAs that they regulate in any manner, but they recruit other RBPs to do so. With the exception of PARP10, they also contain ADP-ribose binding domains (Vyas et al., 2012) (Figure 2A) suggesting that these PARPs could integrate the duration or strength of RNA binding with ADP-ribose binding. These RNA binding domains have specific characteristics that help define their function:

RNA binding CCCH zinc finger domains contain three conserved cysteines followed by a histidine that is used to coordinate a zinc ion (or other metal ions), leading to structural stabilization of the domain (Hall, 2005). Proteins containing CCCH zinc fingers play important roles in RNA processing and function. GO-term analysis of human CCCH zinc finger containing proteins identifies functions in splicing, RNA processing, translation, RNA export, transcription and RNA degradation (Figure 2B). In general zinc finger domains bind RNA targets with high affinity and specificity. Binding affinity and specificity depends on the amino acid sequence of individual zinc finger domains, the number of domains present, and the distance between them (Wolfe et al., 2000).

CCCH PARPs contain CCCH domains that are each similar in length and of generally high homology (% identity between PARP12 and PARP13 CCCH domains =40%; PARP12 and PARP7 =36%; PARP13 and PARP7 =31%). PARP12 and PARP13 contain four CCCH domains, and PARP7 only one. Figure 2A shows a schematic of the CCCH PARP sequence alignment with the relevant domains. So far, PARP13 is the only CCCH PARP with proven RNA binding capability (Chen et al., 2012b; Guo et al., 2004). However, given the sequence similarity between the CCCH domains of PARP12 and PARP13, it is likely that PARP12 also binds RNA. In contrast the presence of only one CCCH domain in PARP7 could limit the protein's ability to bind RNA with high affinity and/or specificity.

The structure of the four tandem PARP13 CCCH domains has been solved (Chen et al., 2012b). Experimental data suggests that a dimer of two PARP13 molecules binds to one RNA molecule. Each of the PARP13 monomers contains two RNA binding cavities formed by the four tandem CCCH domains. Together these two cavities shape a structural feature that, according to molecular modeling predictions, binds specific tertiary structures of RNA rather than linear sequence motifs. This suggests that binding of RNA to PARP13 is complex, and given the homology and the similar spacing of the PARP12 CCCH domains to those of PARP13, it is possible that PARP12 binds RNA in a similarly complex manner.

RNA recognition motifs (RRMs) consist of two alpha helices and four antiparallel beta strands. Several conserved aromatic residues protrude out of the beta strands and are essential for RNA recognition, although some exceptions exist (Daubner et al., 2013). In general, the presence of multiple RRMs is required to confer high sequence specificity and binding affinity (Pérez et al., 1997), however some RBPs that contain only one RRM can also bind RNA with good sequence specificity. Both PARP14 and PARP10 contain just one RRM (Vyas et al., 2012; Yu et al., 2005).

The RRM is one of the most abundant RNA binding domains, with a broad spectrum of target RNAs and sequences (Daubner et al., 2013). In addition to binding RNA, RRM domains can also promote protein-protein binding interactions and their presence in a protein does not necessarily identify it as an RNA binding protein. RRMs recognize multiple RNA structures including single stranded RNA, stem loops or RNA caps. Proteins containing RRM domains function in a variety of pathways, including RNA splicing and decay. This role in mRNA decay is consistent with a newly described function for PARP14 in the regulation of tissue factor mRNA stability (Iqbal et al., 2014). Potential mRNA regulatory functions for PARP10 have not been identified, however the PARP10 RRM appears to contribute to the protein's pro-apoptotic activity upon overexpression (Herzog et al., 2013).

The SAP domain contains several charged and hydrophobic residues that could be used to bind nucleic acids, but the precise mechanism of binding to RNA or DNA is unknown. This domain is primarily associated with DNA binding, although RNA binding has also been reported (Castello et al., 2012; Yang et al., 2006). Many SAP domain containing proteins were identified as RBPs through a proteomics approach (Castello et al., 2012), however only Eri1, an exonuclease involved in RNA processing (Thomas et al., 2014), and PARP2 (Léger et al., 2014) have been proven to bind RNA.

### RBPs as targets of ADP-ribosylation

Various proteomic datasets are available that identify cellular targets of ADP-ribosylation. Given the manner in which the purifications were performed, some of these analyses cannot distinguish between ADP-ribosylated proteins and those that bind to ADP-ribose (Daniels et al., 2014; Gagné et al., 2008; Jungmichel et al., 2013; Zhang et al., 2013). Techniques used include affinity purification with poly(ADP-ribose) binding domains (Jungmichel et al., 2013), immunoprecipitations with anti-poly(ADP-ribose) antibodies (Gagné et al., 2008) or chemical purification of ADP-ribose (Daniels et al., 2014; Zhang et al., 2013). The techniques used by Jungmichel et al. and Gagne et al. likely identify both poly(ADP-ribose)-binding proteins and targets of poly(ADP-ribosyl)ation, whereas Daniels et al. and Zhang et al. identify ADP-ribosylated proteins. Although these studies involve diverse approaches for sample preparation, one consistent result is the enrichment of RBPs, demonstrating a strong connection between PARP and RNA biology (Figure 2C).

Several categories of RNA regulatory proteins were enriched in each analysis suggesting that PARPs play different roles in RNA regulation. These include RNA metabolism (Jungmichel et al., 2013), mRNA processing (Zhang et al., 2013), mRNA metabolism, RNA splicing, and protein synthesis (Gagné et al., 2008). Further evidence comes from a recent meta-analysis comparing several proteomic datasets of ADP-ribosylated/ADP-ribose binding protein to proteomic datasets of RNA granule proteins (Kato et al., 2012). This study by Leung (Leung, 2014) found a very significant overlap, suggesting that ADP-ribose and/or PARPs could play roles in the nucleation, assembly, maintenance and function of RNA granules (Leung, 2014). Thus proteomic analyses of ADP-ribosylated/ADP-ribose binding proteins strongly suggest that PARPs function in multiple aspects of RNA metabolism. It should be noted that one potential limitation of these studies is that they were performed under stress conditions that activate PARP1. Similar analyses should be repeated under non-stress conditions and under RNase and RNase-free conditions to identify specific interactions that could result from RNA-RBP binding.

### PARPs and RNA processing in the nucleus

Pre-mRNAs synthesized during transcription must undergo splicing in order to remove non-coding introns prior to export to the cytoplasm (Black, 2003). These pre-mRNAs are also processed by addition of a 5′ methyl guanine cap and a 3′ poly(A) tail, both of which stabilize the mRNA (Hocine et al., 2010; Moore and Proudfoot, 2009). PARPs modify multiple components of the splicing machinery (Jiang et al., 2010; Prasad et al., 1994), and a study suggests that PARP1 regulates the activity of the poly(A) polymerase (PAP) via poly(ADP-ribosyl)ation (Di Giammartino et al., 2013). This PAP regulatory function was discovered during heat shock, (and is therefore discussed in the stress dependent RNA regulatory section), however it is possible that PARP1 also regulates PAP and poly(A) tail synthesis during non-stress conditions.

PARPs also play important roles in rRNA biology. Ribosomal RNAs (rRNAs) are key enzymatic and structural components of the ribosome. rRNA genes are transcribed as a 45S precursor that is processed into three species of mature rRNA (28S, 18S and 5.8S) (Boisvert et al., 2007). rRNA synthesis and processing occurs in the nucleolus, where ribosomal subunits are assembled prior to export to the cytoplasm (Boisvert et al., 2007; Tschochner and Hurt, 2003).

### Regulation of splicing

One major component of nuclear pre-mRNA regulation by PARPs involves modulation of hnRNP – ribonucleoprotein complexes involved in multiple steps of RNA processing (Chaudhury et al., 2010). These include pre-mRNA splicing and mRNA export to the cytoplasm (Chaudhury et al., 2010). Binding of some hnRNPs to non-processed RNA also acts as an inhibitory signal to prevent export to the cytoplasm (Kim et al., 2005; Nakielny and Dreyfuss, 1996). In general, regulation of splicing by hnRNPs requires binding to specific pre-mRNA sequences to direct the splicing machinery towards specific targets.

**Figure 3:**
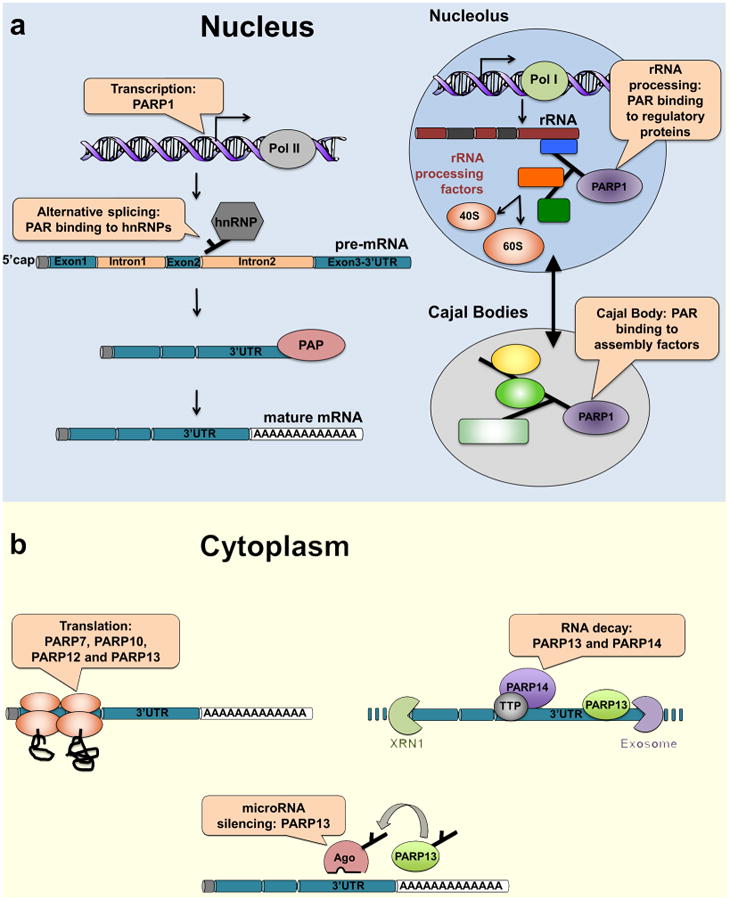
RNA regulation by PARPs during normal conditions (a) PARP1 mediated poly(ADP-ribosyl)ation of histones results in chromatin relaxation and increased accessibility for transcription. During maturation of pre-mRNA, components of the splicing machinery are ADP-ribosylated. The nucleolus, a nuclear structure mainly composed of RNA and RNA binding proteins, is held together by a dense meshwork of poly(ADP-ribose) generated by PARP1. This keeps the components involved in ribosome biogenesis in close proximity to one another and facilitates assembly. In addition, poly(ADP-ribosyl)ation is also required for the shuttling of protein components between the nucleolus and Cajal bodies. (b)Cytoplasmic RNA regulation by PARPs. PARP7, PARP10, PARP12 and PARP13 are involved in translation inhibition, e.g. by ADP-ribosylation of the elongation factor EF2 and ribosomal proteins. PARP13 and PARP14 promote degradation of specific transcripts by targeting these transcripts to the cellular RNA decay machinery. PARP13 can also inhibit microRNA mediated mRNA silencing.

Several hnRNPs (A1, A2/B1, A3, C1/C2, E1, G, H, K, L, M and U) bind to poly(ADP-ribose) or PARP1 (Gagné et al., 2003; Ji and Tulin, 2009, 2013; Kostka and Schweiger, 1982) (Figure 3a). Binding to poly(ADP-ribose) inhibits binding of the hnRNP to target RNAs (Ji and Tulin, 2009, 2012). Other splicing proteins are also either targets of poly(ADP-ribosyl)ation or bind directly to PARPs. These include splicing factor 3A subunit 1 (SF3A1), splicing factor 3B subunit 1 (SF3B1), splicing factor 3B subunit 2 (SF3B2) (Isabelle et al., 2010) and alternative-splicing factor 1/splicing factor 3 (ASF/SF2) (Malanga et al., 2008). The function of poly(ADP-ribose) binding, binding to PARPs, and ADP-ribosylation of these splicing factors is not well understood. More work is required to understand the functional and mechanistic implications on splicing, gene expression, and ultimately on the cell.

### rRNA regulation in the nucleus

Under non-stress conditions, the majority of PARP1 and nuclear poly(ADP-ribose) is enriched in the nucleolus (Kawashima and Izawa, 1981; Leitinger and Wesierska-Gadek, 1993; Rancourt and Satoh, 2009) (Figure 3a). PARP1 knockdown or inhibition via small molecules results in disassembly of the nucleolus, accumulation of rRNA intermediates, and decreased polysome assembly in the cytoplasm (Boamah et al., 2012). The details are so far unclear, however the mechanism appears to involve direct binding of nucleolar proteins to poly(ADP-ribose) that is synthesized by PARP1 localized at the nucleolus. These proteins, that include fibrillarin, AJ1, nucleolin and nucleophosmin, become mislocalized to the cytoplasm when PARP1 function is disrupted (Boamah et al., 2012; Meder et al., 2005). Thus PARP1 appears to regulate nucleolar structure and rRNA processing, and therefore could be important for the efficient assembly of functional ribosomes (Boamah et al., 2012; Boisvert et al., 2007). PARP1 also mediates the shuttling of proteins between the nucleolus and Cajal bodies, nuclear structures where rRNA and splicing factors are processed (Kotova et al., 2009).

PARP1 also regulates rRNA transcription by binding to TTF-1 interacting protein 5 (TIP5), a component of the nucleolar remodeling complex (NoRC) (Guetg et al., 2012). It does so through the noncoding promoter-associated RNA (pRNA). Within this complex, PARP1 becomes active and ADP-ribosylates chromatin as well as TIP5, which leads to transcriptional silencing and the formation of silent rDNA chromatin. PARP1 is therefore an essential factor in nuclear RNA regulatory processes at multiple levels – from modulating chromatin in order to regulate transcription to maintaining nucleolar architecture in order to facilitate rRNA production (Boamah et al., 2012; Guetg et al., 2012).

### Cytoplasmic regulation of mRNA

Once transported to the cytoplasm, mature mRNAs undergo several fates that together determine the amount of transcripts available for translation. In addition to being directly translated into proteins, mRNAs can be degraded through RNA decay, or silenced via microRNA repression. Posttranscriptional regulation of mRNA functions as a critical layer of gene expression fine-tuning (Glisovic et al., 2008), and cytoplasmic PARPs play important roles in the regulation of each of these steps.

### Regulation of translation

Translation of mRNA to protein by the ribosome involves several steps. These include translation initiation, when a complex of proteins assembles onto the 5′ UTR of the mRNA to initiate ribosome assembly, and elongation, when the already assembled ribosome must be translocated to the next codon (Jackson et al., 2010). Both of these steps can be regulated by PARPs.

Canonical mechanisms of translation initiation involves recognition of the 5′ cap of the mRNA, whereas internal ribosomal entry site (IRES) mediated translation initiation uses specific RBPs to target the ribosome to the mRNA independent of the 5′ cap (Hellen and Sarnow, 2001; Jackson et al., 2010; Spriggs et al., 2010). One of these RBPs is hrp38 (the Drosophila ortholog of human hnRNPA1), which recruits ribosomes to an IRES in the DE-Cadherin mRNA to promote translation in Drosophila. Hrp38 binding to the 5′ UTR of DE-Cadherin can be blocked by non-covalent poly(ADP-ribose) binding, resulting in a disruption of ribosome recruitment to the IRES and thus decreased translation of the transcript (Ji and Tulin, 2012).

Mono(ADPribosyl)ation of elongation factor 2 (EF2), a GTPase involved in ribosome translocation, results in the inhibition of protein synthesis (Iglewski et al., 1984; Lee and Iglewski, 1984; Sitikov et al., 1984a, 1984b) (Figure 3b). Treatment with the cytokine Interleukin-1 beta (IL1β) increases EF2 mono(ADPribosyl)ation via unknown mechanisms, potentially to fine-tune translational activity during inflammation (Jäger et al., 2011). Interestingly, a similar mechanism of translation inhibition is employed by bacterial toxins such as diphtheria toxin and exotoxin A, distant relatives of PARPs. These toxins modify EF2 with mono(ADP-ribose) using NAD^+^ as substrate, thereby inhibiting protein synthesis (Lee and Iglewski, 1984). The mechanism of this inhibition is unclear but could include decreased translocation, reduced GTPase activity of EF2 and reduced binding to rRNA, ribosomes or GTP upon mono(ADP-ribosyl)ation (Chen et al., 2012a)

Several ribosomal proteins have been identified as PARP binding proteins, although the functional relevance of this binding is not understood. PARP1 has been shown to bind to the large ribosomal proteins L13, L14, L18a, L21, L22, L23a and L30, (Isabelle et al., 2010; Koyama et al., 1999) as well as the small ribosomal subunit protein S3a, S4, S6 and S13 (Isabelle et al., 2010; Song et al., 2002). PARP1, PARP2 and PARG (a poly(ADP-ribose) hydrolyzing enzyme) show strong enrichment for binding to proteins involved in RNA metabolism (Isabelle et al., 2010), and PARP12 binds to a number of ribosomal proteins and translation initiation and elongation factors (Atasheva et al., 2014). Furthermore, PARP12 overexpression results in translation repression, suggesting that PARP12 could have a regulatory function in translation. However overexpression of PARP12 results in stress granule assembly which itself leads to translation repression (Leung et al., 2011; Welsby et al., 2014).

### RNA decay

A key component of the regulation of gene expression involves mRNA decay. The balance between transcription and the mRNA half-life or decay rate determines the steady-state concentrations of mRNAs available for translation in the cytoplasm. mRNA decay is a process regulated by the activity of specific proteins: 5′ to 3′ decay is mediated by the exonuclease XRN1, and 3′ to 5′ decay by the exosome complex (reviewed in (Schoenberg and Maquat, 2012)). Removal of the 5′ cap by the decapping enzymes DCP1 or DCP2 and removal of the poly(A) tail by deadenylases such as CCR4-NOT, PARN or PAN normally precedes hydrolysis (Coller and Parker, 2004; Schoenberg and Maquat, 2012). Cis-acting elements encoded within the mRNA mediate binding to RNA-regulatory proteins and determine its stability, including destabilizing sequences such as AU-rich elements located in the 3′ UTR (Barreau et al., 2005).

PARP13 destabilizes specific mRNAs by targeting them for RNA decay (Figure 3b) (Guo et al., 2007; Todorova et al., 2014; Zhu et al., 2011). PARP13 binds to cellular mRNA and knockdown or knockout of PARP13 results in a major misregulation of the transcriptome (Todorova et al., 2014). Functional analysis of these misregulated mRNAs revealed an enrichment of signal peptide encoding sequences that are targeted for translation at the endoplasmic reticulum, consistent with the ER localization of PARP13.1, one isoform of PARP13. One major target of PARP13 regulation is TRAILR4 mRNA, that encodes for a member of the TRAIL (TNF-related apoptosis-inducing ligand) receptor family (Todorova et al., 2014). Members of this family bind to TRAIL, a cytokine released by immune cells that has been shown to preferentially induce apoptosis in cancer cells (LeBlanc and Ashkenazi, 2003). TRAILR4 is unable to induce cell death upon TRAIL binding, but rather sequesters TRAIL away from the proapoptotic TRAIL receptors (Mérino et al., 2006). PARP13 binds to the TRAILR4 3′UTR with its CCCH domain and promotes its degradation through the exosome. Regulation of TRAILR4 expression plays an important role in the cellular response to TRAIL, and increased TRAILR4 mRNA levels due to PARP13 deficiency protect cells from TRAIL induced cell death (Todorova et al., 2014).

PARP14 also regulates mRNA stability (Figure 3b). It destabilizes tissue factor (TF) mRNA, which encodes a protein involved in blood coagulation. PARP14 interacts with Tristetraprolin (TTP) and forms a complex that binds to the 3′ UTR of TF mRNA, thereby promoting its degradation (Iqbal et al., 2014). Consistent with a role in the regulation of TF expression, mice deficient in PARP14 exhibit increased TF RNA and TF protein levels. Other PARP14 regulated transcripts have not been identified, however PARP14 does not appear to be a general regulator of TTP function since TNFα, an established target of TTP, is not regulated by PARP14 (Iqbal et al., 2014).

### microRNA regulation by PARPs

MicroRNA silencing is another mechanism used to regulate cytoplasmic levels of translatable mRNAs. Once loaded into the RNA Induced Silencing Complex (RISC), microRNAs bind to target mRNAs, leading to the translational repression and decay of these target mRNAs (Bartel, 2009). Argonaute proteins are critical components of the RISC complex and bind directly to microRNAs and their mRNA targets (Meister, 2013). Each member of the family has been identified as a target of poly(ADP-ribosyl)ation (Leung et al., 2011). Modification of Ago2 with poly(ADP-ribose) reduces its silencing activity possibly by decreasing the binding of Ago2/microRNA complex to target mRNA (Figure 3b). Several PARPs are involved, including CCCH PARP12 and PARP13 and the non-RNA binding PARP5a. PARP13 is essential in this process and appears to be important for targeting Ago2 for poly(ADP-ribosyl)ation despite being enzymatically inactive itself (Leung et al., 2011; Seo et al., 2013).

## RNA regulatory functions during stress

PARPs play important roles in the regulation of RNAs during cellular stresses such as DNA damage, oxidative stress, hypoxia or viral infection (Vyas and Chang, 2014). In general, the manner in which PARPs regulate RNAs during stress is very similar to regulation under non-stress conditions.

### Stress functions in the cytoplasm

Cytoplasmic stressors including hypoxia, oxidative stress, and heat shock, among others, lead to global translational repression. This repression is due to sequestration of mRNAs to cytoplasmic stress granules, structures that contain stalled translation initiation complexes, RNA and RBPs (Anderson and Kedersha, 2008). RBPs that localize to stress granules generally contain low complexity regions and have the propensity to aggregate under the right conditions (Kedersha et al., 2013). In addition, ADP-ribosylation activity in the cytoplasm increases during cytoplasmic stress, and key RBPs are poly(ADP-ribosyl)ated including Ago proteins, TIA1 and G3BP (Isabelle et al., 2012; Leung et al., 2011).

PARP5a, PARP12, PARP13, PARP14 and PARP15 as well as poly(ADP-ribose) and PARG are enriched in stress granules, and modulation of poly(ADP-ribose) levels by overexpressing or depleting PARPs or PARGs affects the assembly, maintenance and disassembly of these structures (Leung et al., 2011). The catalytic activity of PARP12 is important for inducing SG assembly upon its overexpression (Welsby et al., 2014), while the RNA binding domains of both PARP12 and PARP13 are required for their localization to stress granules (Lee et al., 2013; Todorova et al., 2014; Welsby et al., 2014).

**Figure 4:**
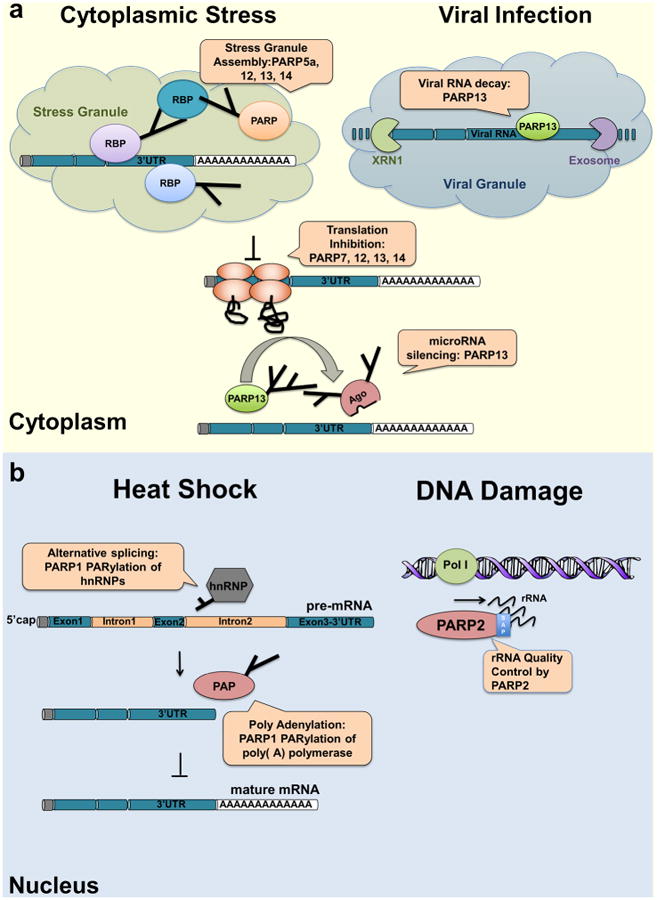
RNA regulation by PARPs during stress conditions (a) Cytoplasmic stress induces stress granule assembly mediated by PARP5a, PARP12, PARP13 and PARP14. microRNA mediated silencing is relieved upon cytoplasmic stress or viral infection, a process which requires PARP13 function. PARP7, PARP12, PARP13 and PARP14 directly inhibit translation in response to stress or upon viral infection. Viral RNA is additionally targeted to the RNA decay machinery by PARP13. (b)During heat shock PARP1 regulates splicing by recruiting hnRNPs to poly(ADP-ribosyl)ated proteins. This results in the dissociation of the hnRNPs from their target mRNAs. Heat shock activated PARP1 also mediates poly(ADP-ribosyl)ation of poly(A)polymerase (PAP) resulting in decreased polyadenylation activity of the protein. RNAs lacking poly(A) tails fail to be exported to the cytoplasm and are degraded. During DNA damage, PARP2 binds to accumulated rRNA through its SAP domain activating its enzymatic activity.

Viral infection induces the assembly of antiviral stress granules, structures that are highly similar to cytoplasmic stress granules and contain many of the same biochemical components (Onomoto et al., 2014), including PARP12 and PARP13 (Atasheva et al., 2014; Lee et al., 2013). Interestingly the expression of PARP13 and the PARP13.2 isoform are upregulated during viral infection in an interferon dependent manner (Hayakawa et al., 2011; Welsby et al., 2014) (Figure 4a). During the immune response, these antiviral granules inhibit viral translation to prevent replication (Lloyd, 2013; Rozelle et al., 2014). They are enriched in components of the RNA decay machinery including RCK, PMR1, TTP, KSRP and Xrn1 (Onomoto et al., 2014).

PARP13 was initially discovered as an antiviral factor in a cDNA expression screen to identify suppressors of murine leukemia virus (MLV) infection (Gao et al., 2002) (Figure 4a). It was subsequently determined that PARP13 is active against several, but not all RNA viruses including MLV, SINV, EBOV, HIV, and the RNA replication intermediates of Hepatitis B DNA virus (Bick et al., 2003; Mao et al., 2013; Müller et al., 2007; Zhu et al., 2011). PARP13 binds to viral RNA through its CCCH domain, but the binding site varies in different viruses (the 3′ LTR in MLV, terminal redundancy sequences in HBV, the filovirus L protein transcript in EBOV, various fragments within the SINV genome and the 5′ UTR of several spliced HIV RNAs) and no sequence similarity among these regions has been identified (Guo et al., 2004; Mao et al., 2013; Müller et al., 2007; Zhu et al., 2011). It interacts directly with exosome components RRP46/EXOSC5, RRP42/EXOSC7 and with the deadenylase PARN, and recruits DCP1, DCP2 and XRN1 indirectly via DDX17 (Guo et al., 2007; Zhu et al., 2011). Thus PARP13 appears to recruit decay factors in an RNA sequence specific manner to mediate 5′-3′ and 3′-5′ decay of viral RNAs, similar to its role in regulating cellular RNA decay.

PARP13-mediated regulation of microRNA activity also occurs during the antiviral response (Figure 4a). Many antiviral genes have cytotoxic effects, therefore their expression is tightly controlled under non-infection conditions partially through microRNA activity (Seo et al., 2013). Viral infection results in increased poly(ADP-ribosyl)ation of Ago2 and PARP13, resulting in the inactivation of the RNAi machinery and a derepression of antiviral gene expression (Seo et al., 2013). This results in a robust induction of the antiviral response.

Translation of viral proteins can also be inhibited by RNA binding PARPs including PARP7, PARP12, and PARP13. PARP12 binds to ribosomal proteins in polysome containing, actively translating fractions upon infection with the RNA virus Venezuelan equine encephalitis virus (VEEV) resulting in translation inhibition (Atasheva et al., 2012, 2014). This function of PARP12 requires RNA binding. A similar role has been identified for PARP13 (Bick et al., 2003) that also involves binding of viral RNA and binding of PARP13 to the initiation factor eIF4A (Zhu et al.,2011). A requirement for RNA binding for PARP7 function in the inhibition of viral RNA translation has not been identified (Atasheva 2012, 2014).

### Stress functions in the nucleus

One of the best studied PARP-mediated nuclear stress responses is heat shock. The heat shock response involves changes in transcription, but also altered processing of RNAs in the nucleus (Richter et al., 2010). In general, transcription of most genes is reduced in order to minimize the protein-folding load of the cell during stress. Nuclear PARPs 1 and 2 play important roles in the nuclear stress responses. PARP1 regulates the transcriptional response to heat shock and is also involved in the processing of heat shock response genes.

One important PARP1 function during the nuclear stress response is poly(ADP-ribosyl)ation of poly-A-polymerase (PAP) (Di Giammartino et al., 2013). Modification of PAP results in reduced 3′ mRNA processing (Figure 4b). Binding and modification of PAP with poly(ADP-ribose) by PARP1 leads to dissociation of PAP from target mRNAs, decreased poly-adenylation, and repression of mature mRNA synthesis (Di Giammartino et al., 2013). The net result is decreased synthesis of new protein, reducing the protein-folding load in heat-shocked cells. Inefficient 3′ processing and poly-adenylation of mRNAs has also been implicated in the inhibition of mRNA export to the cytoplasm (Huang and Carmichael, 1996; Libri et al., 2002; Vinciguerra and Stutz, 2004). Therefore PARP1 appears to inhibit a key step in Mrna processing with important consequences for the cell and its ability to properly respond to stress (Di Giammartino et al., 2013). It is not known if PARP1 modifies PAP under non-stress conditions, however the possibility remains that PARP1 also functions as a general posttranscriptional regulator of cellular mRNAs by regulating PAP function.

The increased PARP1 activity upon heat shock also regulates splicing by altering the RNA binding dynamics of hnRNPs (Ji and Tulin, 2009) (Figure 4b). In Drosophila, the hnRNPs hrp38 and squid normally promote splicing of specific target RNAs by binding to exonic splicing enhancer sites (ESE). They can also prevent splicing at specific exons by binding to intronic splicing silencing sites (ISS), resulting in exon skipping (Ji and Tulin, 2009). Upon heat shock and the resulting increase in nuclear poly(ADP-ribosyl)ation, both proteins bind to poly(ADP-ribose) and dissociate from their target mRNAs. The withdrawal of hrp38 and squid from ESE sites then prevents intron splicing, whereas removal from ISS sites prevents exon skipping and alternative splicing (Ji and Tulin, 2009). This differential splicing could therefore be an important response to heat shock and suggests that similar mechanisms could exist under non-stress conditions.

Another type of stress, long known to involve ADP-ribosylation, is DNA damage. Simultaneous treatment with the transcription inhibitor Actinomycin D during DNA damage results in accumulation of rRNA intermediates and increased PARP2 dependent poly(ADP-ribose) synthesis (Léger et al., 2014) (Figure 4b). The increased production of poly(ADP-ribose) by PARP2 is mediated by RNA binding to its SAP domain and only occurs upon simultaneous genotoxic stress (Léger et al., 2014). As transcription inhibition alone is insufficient to activate PARP2, this suggests that PARP2 must be pre-activated in order to be responsive to RNA or RNA accumulation in the nucleus. Interestingly, both rRNA and mRNA activated PARP2 enzymatic activity *in vitro* and RNA damage was not required to induce this increased poly(ADP-ribosyl)ation activity. These results suggest that PARP2 could function as a sensor for RNA accumulation in the nucleus (Léger et al., 2014) and could have important consequences for the stressed cell since inhibition of RNA export to the cytoplasm is characteristic of certain stress responses (Hopper et al., 1996).

## Conclusion

RNA regulation is an exciting area of PARP biology that is just starting to hit its stride. Regulation of RNA processing by PARPs occurs at nearly all of the key regulatory steps, and its importance as a regulatory mechanism for gene expression during non-stress conditions, as well as during cell stress has become apparent. In general PARPs regulate RBP function either by directly modifying them with ADP-ribose, or by producing poly(ADP-ribose) that sequesters the RBPs through non-covalent binding interactions, decreasing their ability to bind RNAs. Nuclear and cytoplasmic regulation of RNAs by PARPs have many similarities, however one key difference is that cytoplasmic regulation involves RNA-binding PARPs that bind to and regulate RNAs in a sequence specific manner, whereas nuclear PARPs do not.

The challenge ahead is to identify the specific mechanisms by which PARPs regulate RNA and RBP function. This will allow us to study equally important questions including potential coordination of PARPs in the regulation of key RNA regulatory steps and identification of common mechanisms of regulation, and to address the importance of RNA regulation by PARPs in cells, model organisms, and its potential importance in human disease. As a family of proteins PARPs have been shown to be druggable and PARP1 inhibitors are promising therapeutics for the treatment of cancer (Lord et al., 2015). Targeting PARPs involved in RNA regulation could have additional therapeutic indications including the treatment of autoimmune or inflammatory diseases, where post-transcriptional regulation of RNAs is often misregulated (Anderson, 2008).

Recent technological innovations in PARP biology, RNA analysis, and gene editing should lead to rapid progress in elucidating the function of the different RNA binding PARPs. These include the ability to identify specific amino acid residues on target proteins that are modified by ADP-ribose, allowing us to more closely examine the mechanistic consequences of ADP-ribosylation (reviewed recently in Vivelo and Leung 2014). They also include technical advances in the RNA field such as CLIP-seq (Hafner et al., 2010; Licatalosi et al., 2008) that will facilitate the identification of RNAs bound to RNA-binding PARPs, and allow us to determine the effects of ADP-ribosylation or ADP-ribose binding on the RNA binding specificity of RBPs. In addition, high-throughput sequencing of cells with targeted deletion or targeted mutation of specific PARPs generated using gene editing techniques (Hsu et al., 2014) will allow us to identify the transcripts that are targets of PARP regulation. With these tools in hand, and new tools that will surely be developed, the future of this area of PARP biology is indeed bright.
